# Opioid reduction for patients with chronic pain in primary care: systematic review

**DOI:** 10.3399/BJGP.2021.0537

**Published:** 2022-02-08

**Authors:** Loes de Kleijn, Julie Rønne Pedersen, Hanneke Rijkels-Otters, Alessandro Chiarotto, Bart Koes

**Affiliations:** Department of General Practice, Erasmus Medical Centre, University Medical Centre Rotterdam, the Netherlands.; Department of Sports Science and Clinical Biomechanics, University of Southern Denmark, Odense, Denmark.; Department of General Practice, Erasmus Medical Centre, University Medical Centre Rotterdam, the Netherlands.; Department of General Practice, Erasmus Medical Centre, University Medical Centre Rotterdam, the Netherlands.; Department of General Practice, Erasmus Medical Centre, University Medical Centre Rotterdam, the Netherlands, and Department of Sports Science and Clinical Biomechanics, University of Southern Denmark, Odense, Denmark.

**Keywords:** chronic pain, family practice, opioid, opioid epidemic, patient care, systematic review

## Abstract

**Background:**

Long-term opioid treatment in patients with chronic pain is often ineffective and possibly harmful. These patients are often managed by GPs who are calling for a clear overview of effective opioid reduction strategies for primary care.

**Aim:**

To evaluate effectiveness of opioid reduction strategies applicable in primary care for patients with chronic pain on long-term opioid treatment.

**Design and setting:**

Systematic review of controlled trials and cohort studies performed in primary care from inception date to 15 January 2021.

**Method:**

Literature search conducted in EMBASE, MEDLINE, Web of Science, Cochrane Central Register of Controlled Trials, CINAHL, Google Scholar, and PsycINFO. Studies evaluating opioid reduction interventions applicable in primary care among adults on long-term opioid treatment for chronic non-cancer pain were included. Risk of bias was assessed using the Cochrane risk-of-bias tool (version 2) (RoB 2) or the Risk of bias in non-randomized studies — of interventions (ROBINS-I) tool. Narrative synthesis was performed because of clinical heterogeneity in study designs and types of interventions.

**Results:**

In total, five randomised controlled trials (RCTs) and five cohort studies were included (*N* = 1717, range *n* = 35 to *n* = 985) exploring various opioid reduction strategies. Of these, six studies had high/critical RoB, three moderate RoB, and one low RoB. Three cohort studies: investigating a GP-supervised opioid taper (critical ROBINS-I), an integrative pain treatment (moderate ROBINS-I), and group medical visits (critical ROBINS-I) demonstrated significant between-group opioid reduction.

**Conclusion:**

Results carefully point in the direction of a GP supervised tapering and multidisciplinary group therapeutic sessions to reduce long-term opioid treatment. However, because of high risk of bias and small sample sizes, no firm conclusions can be made demonstrating the need for more high-quality research.

## INTRODUCTION

Where opioids once were the panacea to chronic non-cancer pain (CNCP), today the negative effects of opioids are well known. Short-term side effects of opioids include, among others, sedation and respiratory depression.^1^ Opioids, also when prescribed by a doctor, are addictive in nature and may lead to tolerance, dependence, and addiction.^2^^–^^4^ Various observational studies suggest a dose-dependent association between long-term opioid treatment (LTOT) and an increased risk of myocardial infarction, fractures, falls, and even all-cause mortality.^5^^,^^6^ In addition, a growing amount of evidence indicates no difference in short-term effectiveness of opioid and non-opioid therapy for CNCP.^5^ Research on long-term effectiveness of opioids on CNCP is still scarce, but limited available evidence suggests merely a weak effect of LTOT on pain relief in CNCP.^7^^–^^9^ Considering these serious harms and limited effectiveness, clinical guidelines on the management of CNCP no longer recommend opioid treatment.^8^^,^^10^^–^^12^

Key to turning the tide against the opioid epidemic is to reduce LTOT in patients with CNCP. In the US the estimated prevalence of LTOT among patients with CNCP ranges between approximately 1 and 9%.^13^ GPs have by default become responsible for reducing LTOT because of waiting lists at addiction clinics and pain centres. Yet, qualitative research among GPs report that GPs around the world feel ill equipped to reduce opioid use in these patients.^14^^,^^15^ In other words, GPs playing a pivotal role in the opioid epidemic call for a clear overview of effective opioid reduction strategies that they can safely use in primary care.

Recently, three systematic reviews were published on effectiveness of opioid reduction strategies in CNCP;^16^^–^^18^ two of these reviews searched the literature up to 2017, whereas several trials have been published in more recent years.^19^^–^^24^

In addition, all three reviews looked at all available opioid reduction strategies and, thus, also included strategies that are not applicable in primary care. The present systematic review was specifically aimed at evaluating the most recent evidence on effectiveness of opioid reduction strategies for patients with CNCP on LTOT that are applicable in primary care.

## METHOD

This systematic review is reported according to the Preferred reporting items for systematic reviews and meta-analyses (PRISMA) guidelines.^25^ The review protocol was pre-registered in PROSPERO (CRD42021236399).

**Table:** How this fits in

Though GPs are key players in tackling the opioid crisis, they feel ill equipped to reduce long-term opioid treatment (LTOT) in patients with chronic pain. This systematic review aimed to evaluate the effectiveness of opioid reduction strategies for primary care. The results from this systematic review suggest that multidisciplinary GP-supervised and multidisciplinary group-based therapeutic sessions may be effective in reducing and discontinuing LTOT. However, because of high risk of bias and small sample sizes, no strong conclusions can be made, demonstrating the need for further high-quality research in this field.

### Data source and search strategy

Searches were carried out in EMBASE, MEDLINE (Ovid), Web of Science Core Collection, Cochrane Central Register of Controlled Trials, CINAHL, Google Scholar, and PsycINFO, from inception date to 15 January 2021 without restriction on language. The complete search strategy is presented in Supplementary Box S1. Backward citation tracking of eligible studies and backward snowballing of recent reviews^16^^–^^18^ were performed.

### Selection of studies

Studies evaluating opioid reduction interventions applicable in primary care among adults on LTOT for CNCP were included. The first and second authors (reviewers) independently screened the articles by title and abstract using eligibility criteria presented in Box 1. All studies deemed eligible by at least one reviewer were read in full for eligibility by the same two reviewers. Where consensus between reviewers was not reached, a third reviewer (fifth author) was consulted.

**Box 1.** Eligibility criteria

**Study inclusion criteria**
Randomised controlled trial or cohort study with control group
Evaluates an intervention aimed to facilitate opioid dose reduction or discontinuation
Performed on patients aged ≥18 years
Performed on patients with chronic non-cancer pain, that is, non-cancer pain lasting>3 months
Performed receiving long-term opioid treatment prescribed by, and legally obtained through, a physician
Performed in primary care or intervention is applicable in primary care
Reports on opioid reduction in mg morphine equivalent daily dose (MEDD)
Published and presented in full text

**Study exclusion criteria**
Includes pregnant patients
Includes patients with an oncological diagnosis
Includes patients receiving palliative treatment
Includes patients with acute or subacute pain, that is, pain lasting <3 months
Includes patients with opioid treatment <3 months

### Data extraction and risk of bias assessment

Study characteristics were extracted by the first author and checked by the second. Outcome data were extracted independently by both the first and second authors using a standardised extraction form. Data extracted included, among others, intervention characteristics; comparison characteristics; primary outcome measures (mean opioid dose in mg morphine equivalent daily dose [MEDD] at baseline and end-of-intervention and/or mean opioid dose change); and secondary outcome measures (opioid discontinuation rates, incidence of adverse events, withdrawal symptoms, physical functioning, measures of mental wellbeing, and overall quality of life). For cohort studies, outcome data at the last available timepoint when still receiving the intervention were extracted and, for studies that did not report outcome at end-of-intervention, outcome data at the next available timepoint were extracted.

The first and second authors independently assessed risk of bias of the included studies using the Cochrane risk-of-bias tool (version 2) (RoB 2) for randomised controlled trials^26^ and Risk of bias in non-randomized studies — of interventions tool (ROBINS-I) for non-randomised studies.^27^ Disagreements between reviewers were solved by consensus.

### Data synthesis

Due to substantial clinical heterogeneity in type of interventions and study designs, though a priori planned, meta-analysis was not possible and a narrative synthesis approach was adopted following the Synthesis without meta (SWiM) analysis guideline.^28^ Effectiveness of interventions is presented as between-group differences and/or (if between-group differences were not available or if the outcome at baseline was significantly different between the groups) as within-group difference. Adjusted analysis correcting for baseline imbalances were presented if these resulted in a different conclusion.

## RESULTS

**Figure 1.**
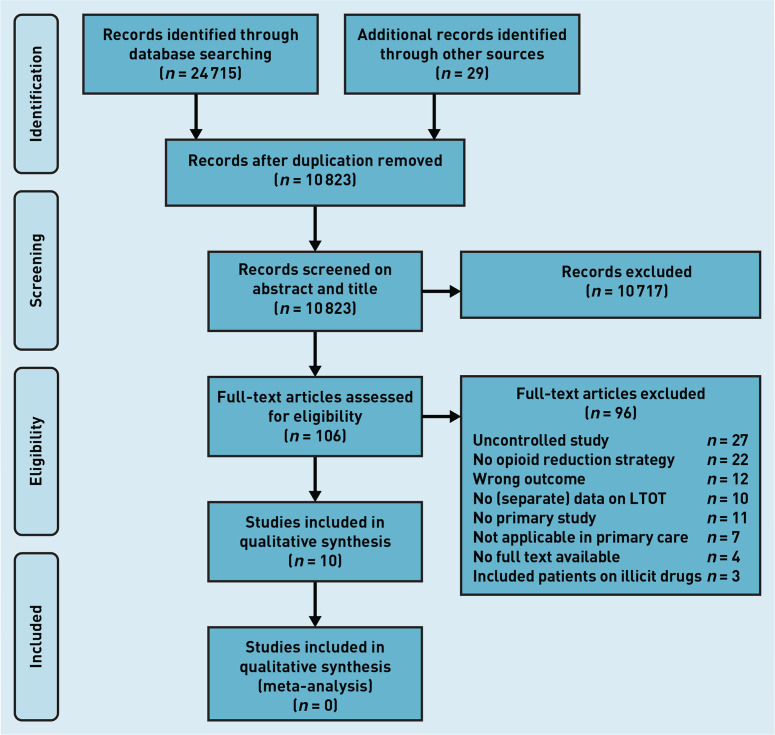
*PRISMA flowchart: selection process for systematic review on opioid reduction for patient with chronic pain in primary care. LTOT = long-term opioid treatment.*

The search initially retrieved 10 823 studies after removing duplicates. Of these, 106 studies were selected based on their abstracts and titles, and read in full. The corresponding authors of seven of these studies were contacted through mail because information on inclusion criteria was missing. All but one author responded to this mail. After review, 10 studies^19^^–^^24^^,^^29^^–^^32^ met the inclusion criteria and were included in this review (Figure 1).

### Study characteristics

The 10 included studies comprised *N* = 1717 (range *n* = 35 to *n* = 985) patients. Five studies^19^^,^^21^^,^^29^^,^^31^^,^^32^ were randomised controlled trials (RCTs) and five studies^20^^,^^22^^,^^23^^,^^24^^,^^30^ were cohort studies. A variety of opioid reduction methods, from tapering protocols to alternative care strategies, were explored. Supplementary Table S1 includes a summary of studies and patient characteristics included in this systematic review.

### Risk of bias

**Figure 2.**
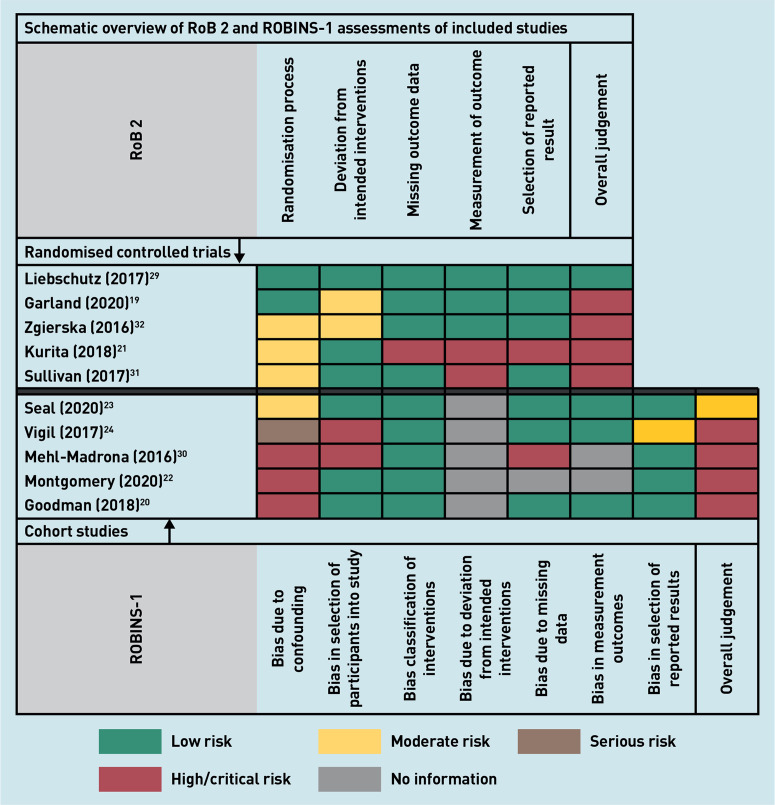
*Schematic overview of RoB 2 and ROBINS-1 assessments of included randomised controlled trials and cohort studies. RoB 2 = Cochrane risk-of-bias tool. ROBINS-1 = Risk of bias in non-randomized studies — of interventions tool.*

One RCT^29^ had low risk of bias, two RCTs^19^^,^^32^ had some concerns because of non-blinding of research participants and research staff, and the two remaining RCTs^21^^,^^31^ had high risk of bias (Figure 2). One cohort study^23^ had moderate risk of bias; the remaining four studies^20^^,^^22^^,^^24^^,^^30^ had a critical risk of bias (Figure 2). Complete RoB 2 and ROBINS-I assessments of the included cohort studies are presented in Supplementary Boxes S2 and S3.

### Narrative summary of results

None of the included RCTs demonstrated a significant between-group difference in opioid dose. Three out of the five relatively small cohort studies,^20^^,^^23^^,^^30^ one with a moderate risk in bias (*n* = 294) and two with a critical risk in bias (*n* = 84 and *n* = 41), demonstrated a significant between-group difference in opioid reduction favouring the experimental intervention groups. The interventions researched by these cohort studies varied from multidisciplinary pain treatment,^23^ group therapeutic sessions,^30^ to an individually tailored taper plan.^20^

### Narrative synthesis per study

A narrative synthesis of the included studies, primary outcomes, and a brief discussion of secondary outcomes is presented below. An overview and thorough explanation of outcomes is presented in Table 1.

**Table 1.** Schematic overview of study results on primary and secondary outcomes

**Study**	**Study design**	***n* (total)**	**Intervention versus control**	**Risk of bias^a^**	**Primary outcome**	**Secondary outcomes**			
					**Opioid dose,^b,c^ mg mean difference (95% CI and/or *P*-value)**	**Discontinuation rate, %^b^ (95% CI and/or *P*-value)**	**Pain^d^ (95% CI and/or *P*-value)**	**Physical function^b^ (*P*-value)**	**Mental wellbeing^b^ (95% CI)**
Liebschutz (2017)^29^	RCT	985	TOPCARE versus electronic decision tool	Low risk	6.5 (*P* = 0.31)	−4.5 (*P*= 0.08)			
Garland (2020)^19^	RCT	95	MORE versus active support group	Some concerns	110.6^e,f^				
Zgierska (2016)^32^	RCT	35	Meditation-cognitive behavioural therapy versus usual care for CNCP	Some concerns	5.7 (−34.3 to 45.7)^g^		0.9 (0.01 to 1.7)^g^	1.9 (−5.5 to 9.3)^g^	−0.4 (−5.2 to 4.4)^g^
Kurita (2018)^21^	RCT	35	Taper off versus usual care	High risk	74.2 (*P*= 0.446)^h^		−0.2 (*P*= 0.968)		
Sullivan (2017)^31^	RCT	35	Taper support intervention versus usual care	High risk	57.91^f,i^		0.68 (0.64 to 2.01; *P*= 0.30)^j^		
Seal (2020)^23^	Cohort	294	Integrative pain team treatment versus usual primary care	Moderate	38.7 mg (*P*= 0.03)^k^				
Vigil (2017)^24^	Cohort	66	Medical cannabis programme versus usual care	Critical	−0.1 mg (*P*= 0.0974)	−37.1 (*P*<0.001)			
Mehl-Madrona (2016)^30^	Cohort	84	Group medical visit versus usual care	Critical	53.7^f,l^	−19 (*P*<0.001)			
Montgomery (2020)^22^	Cohort	47	Battlefield acupuncture versus usual care	Critical	18.85^f^		0 (*P*= 0.15)		
Goodman (2018)^20^	Cohort	41	Individually tailored opioid taper versus treatment in medical pain clinic	Critical	118.26 (23.23 to 213.31; *P*= 0.018)				

a *RCTS were assessed using the RoB 2 tool and cohort studies were assessed using ROBINS-I.*

b *Mean between-group difference comparing control with intervention group at end-of-intervention, unless stated differently.*

c *Measured in mg of morphine equivalent daily dose (MEDD).*

d *Pain severity on a numeric rating scale (NRS).*

e *Measured at 4 weeks after intervention was completed.*

f P-*values or confidence interval were not reported.*

g *This value is a mean change difference comparing control with intervention.*

h *Because of excessive drop-out rate, data assessment was performed at 3.5 months’ follow-up.*

i *In an adjusted analysis for baseline imbalances, the mean between-group difference in MEDD was −42.9 mg (95%CI = −92.42 to 6.62,* P*=0.09).*

j *Numbers based on the adjusted analysis for baseline imbalances.*

k *Of note, participants in the integrative pain team group reported higher opioid misuse rates and were diagnosed more often with an opioid use disorder. In adjusted analysis controlling for these and other baseline imbalances using a mixed-effects linear regression model, the between-group difference remained significant with a mean between-group difference of 38.2, 95%CI = 13.0 to 63.5 mg,* P<*0.01.*

l *The within-group differences comparing opioid dose at end-of-intervention with baseline were significant, mean difference intervention group −49.7 mg MEDD, mean difference control group +14.0 mg MEDD, both with* P<*0.001. Note that, initially, 207 participants attended the intervention, yet the analysis of the intervention’s effect was performed using data from the 42 participants who had attended the intervention for at least 6 months. CI = confidence interval. CNCP = chronic non-cancer pain. MORE = Mindfulness-oriented recovery enhancement. RCT = randomised controlled trial. RoB 2 = Cochrane risk of bias tool (version 2). ROBINS-1 = Risk of bias in non-randomized studies — of interventions tool. TOPCARE = Transforming opioid prescribing in primary care.*

### Description of results in RCTs

#### Liebschutz (2017).^29^

In this cluster RCT (*n* = 985), 1 year of an intervention, Transforming opioid prescribing in primary care (TOPCARE), consisting of nurse care management, electronic registry, academic detailing, and electronic decision tools was compared with 1 year of electronic decision tools only. There was no significant between-group difference in opioid dose at end-of-intervention (mean difference 6.5 mg MEDD, *P* = 0.31). An adjusted regression analysis accounting for baseline imbalances demonstrated a statistically significant lower opioid use in the TOPCARE group (mean difference [standard deviation] 6.8 [SD 1.6] mg MEDD, *P*<0.001). Additionally, a non-statistically significant difference in opioid discontinuation among the groups was reported.

#### Garland (2020).^19^

In this RCT (*n* = 95), Mindfulness-oriented recovery enhancement (MORE), consisting of 8 weekly 2-hour mindfulness group sessions and daily 15-minute mindfulness practices at home, was compared with an active support group in which the group sessions consisted of discussions on chronic pain and opioid reduction. In both groups there was no explicit call made to reduce opioid treatment. The study did not report on opioid dose at end-of-intervention, but at 1 month after intervention. The between-group mean difference was 110.6 mg MEDD in favour of the intervention (confidence intervals and *P*-values not provided). An intention-to-treat analysis using a linear mixed model with an interaction of group and time demonstrated a statistically significant between-group difference (*P* = 0.006).

#### Zgierska (2016).^32^

In this RCT (*n* = 35), patients were randomised to either meditation-cognitive behavioural therapy, consisting of 2-hour weekly group sessions and encouragement in formal mindfulness, or usual care. At the end-of-intervention period, groups did not significantly differ in opioid dose change (mean difference 5.7 mg MEDD, 95% confidence interval (CI) = −34.3 to 45.7). Additionally, a significant between-group change in pain severity was reported in favour of the experimental intervention. There was no significant between-group difference in change in physical function or in perceived stress.

#### Kurita (2018).^21^

In this RCT (*n* = 35), all included participants were (if needed) switched to sustained-release opioid therapy, after which they were randomised to either a taper-off intervention group or usual care. The intervention consisted of motivational talks and weekly or bi-weekly 10% reduction of opioid dose until discontinuation. Because of a large participant drop-out, outcome assessments were performed at 3.5 months. The mean between-group difference in opioid use was not statistically significant (mean difference 74.2 mg MEDD, *P* = 0.446). Additionally, the study reported no significant between-group difference in pain severity.

#### Sullivan (2017).^31^

In this RCT (*n* = 35), participants interested in tapering their opioid dose were randomly assigned to either a taper support intervention or usual care. The intervention consisted of one visit with a physician followed by 17 weekly sessions in cognitive behavioural therapy for chronic pain with a physician assistant followed by weekly dose reduction of 10%. Between-group mean difference was 57.91 mg MEDD in favour of the taper support intervention (confidence interval and *P*-value not reported). Adjusted analysis for baseline imbalances reported a non-significant between-group difference in MEDD (mean difference −42.9 mg MEDD, 95% CI = −92.42 to 6.62, *P* = 0.09) and pain severity.

### Description of results in cohort studies

#### Seal 2020.^23^

In this cohort study (*n* = 294), participants receiving consultations from an integrated pain team, consisting of primary care providers with training in pain management, pain pharmacists, and pain psychologists, were compared with participants receiving usual primary care (moderate ROBINS-I). At 6 months the mean between-group difference in opioid reduction was statistically significant in favour of the experimental intervention (mean difference 38.7 mg MEDD, *P*<0.03).

#### Vigil 2017.^24^

In this cohort study (*n* = 66), participants enrolled in a medical cannabis programme were compared with participants who had declined the offer to enrol in the programme. The difference in opioid dose was non-statistically significant between groups (mean 0.1 mg MEDD, *P* = 0.974). The patients in the cannabis programme were significantly more likely to discontinue their opioid treatment.

#### Mehl-Madrona 2016.^30^

In this cohort study (*n* = 84), participants attending group medical visits (GMVs) at least twice monthly, in which non-pharmacological, complementary, and alternative therapy were encouraged for the treatment of pain, were matched with participants receiving usual care in the same age decile, with same major diagnosis, same sex, and within 25% in mg MEDD (critical ROBINS-I). At end-of-intervention (range 6 months to 2.5 years) a statistically significant mean between-group difference of 53.7 mg MEDD was reported in favour of the intervention (CI and *P*-value were not reported). Additionally, a statistically significant difference in between-group discontinuation rate in favour of the intervention was reported. Differences in pain severity and quality of life were only reported for the intervention group, both demonstrating a statistically significant within-group change.

#### Montgomery (2020).^22^

In this cohort study (*n* = 47), battlefield acupuncture (BFA), a unique five-point auricular acupuncture procedure, was compared with usual care in patients on long-term opioid pain contract. The study reported a non-significant between-group mean difference of 18.85 mg MEDD (CI and *P*-value not provided). Of note, both groups increased opioid dose over the course of the study (mean difference BFA +3.9 mg MEDD, control +8.7 mg MEDD, CI and *P*-values not provided). The study reported no significant difference in pain severity.

#### Goodman (2018).^20^

In this cohort study (*n* = 41), patients had a conversation with their family GP discussing opioid cessation, after which they could choose either individually tailored opioid tapering by their family GP or further pain treatment at a medical pain clinic (critical ROBINS-I). A statistically significant between-group difference in opioid use in favour of the GP-supervised tapering was reported (mean difference 118.26 mg MEDD, 95% CI = 23.23 to 213.31, *P* = 0.018). However, the groups differed significantly in mean opioid dose at baseline with a higher opioid dose in the control group (mean difference 142.15 mg MEDD, 95% CI = 51.69 to 232.62, *P* = 0.005). Within-group difference comparing opioid dose at baseline with opioid dose at end-of-intervention demonstrated a significant reduction in the GP-supervised tapering group (mean difference 14.85 mg MEDD, 95% CI = 5.58 to 24.12, *P* = 0.003) and a non-significant reduction in the control group (mean change 38.74 mg MEDD, 95% CI = −42.88 to 120.368, *P* = 0.324).

## DISCUSSION

### Summary

A total of five RCTS and five cohort studies were included in this systematic review. Studies were generally small and overall risk of bias was high. One RCT was graded low-risk bias^29^ (RoB 2 tool) but none of the cohort studies received this grading (ROBIN-S tool). There were some overarching principles explored: five studies^19^^,^^23^^,^^30^^–^^32^ used psychological interventions as part of the intervention, four studies^19^^,^^23^^,^^30^^,^^32^ explored the effect of therapeutic group sessions, and three studies^20^^,^^21^^,^^31^ looked at opioid tapering. None of the RCTs demonstrated a significant between-group difference in opioid dose. Three out of the five cohort studies^20^^,^^23^^,^^30^ demonstrated a significant between-group difference in opioid reduction favouring the experimental intervention groups.

The results from this systematic review suggest that multidisciplinary GP-supervised and multidisciplinary group-based therapeutic sessions may be effective in reducing and discontinuing LTOT. However, because of high risk of bias and small sample sizes, no strong conclusions can be made, demonstrating the need for further high-quality research in this field.

### Strengths and limitations

Conclusions of this systematic review are not without limitations. A comparison of study results was proposed by extracting between-group difference at end-of-intervention. However, in two studies, by Garland *et al*^19^ and Kurita *et al*,^21^ these results were not provided. In Kurita *et al* (2018),^21^ because of loss of follow-up the extraction timepoint was brought forward, that is, before the end-of-intervention was reached, causing possible bias away from the null. Whereas, in Garland *et al* (2020),^19^ the extraction timepoint was 10 weeks after intervention, creating possible bias towards the null.

The generalisability of findings presented in this review to countries outside of the US is limited, since all but one study was performed in the US. It cannot be simply assumed that effectiveness of strategies for patients with CNCP on LTOT is the same all over the world, especially since the opioid crisis in the US has been found to be very different compared with Europe and the rest of the world.^33^ Moreover, to compare effectiveness equally and objectively among all included studies, 12 studies were excluded as they did not report opioid dose reduction in MEDD. However, though excluded from this review, they might have reported on effective opioid reduction interventions.

Finally, studies were excluded if they were considered not applicable in primary care, which was at the discretion of the first and second reviewers, who based their opinions on the types of strategies that would be applicable in the Netherlands and Denmark. Since primary care services vary from country to country, studies might have been included (or excluded) that could not (or could) have been implemented in the primary care of other countries.

### Comparison with existent literature

This systematic review included one RCT^19^ that was not included in two most recent systematic reviews.^17^^,^^18^ Additionally, four new cohort studies,^20^^,^^22^^–^^24^ which had not been included in the most recent systematic review,^16^ were included. Hence, this review is, to the authors’ knowledge the most up-to-date review discussing intervention effectiveness in studies exploring opioid reduction strategies for patients with CNCP on LTOT applicable in primary care. Moreover, this is the first review that has included only studies on opioid reduction strategies that are applicable in primary care.

Though this review specifically explored reduction strategies applicable in primary care, the overall conclusion is in line with other recent reviews,^16^^–^^18^ namely that no strong conclusions can be made regarding the benefit of opioid reduction strategies for people with CNCP due to overall high risks of bias and small sample sizes. With five new and recent studies identified,^19^^,^^20^^,^^22^^–^^24^ the presented review demonstrates a fast growth of studies exploring opioid reduction strategies.

### Implications for research and practice

This review demonstrates the need for more high-quality research on opioid reduction strategies for patients with CNCP on LTOT. The fact that multiple research protocols for future research in this field were identified while scanning abstracts and titles^34^^–^^37^ can be seen as a step in the right direction. These upcoming studies should build on lessons learned. Future research should include high-quality RCTs, blinded for patients or at least for research personnel, where possible, to reduce risks of bias. In addition, large drop-out rates, demonstrated by some included studies,^19^^,^^21^^,^^30^ can be expected and studies should opt for larger sample sizes than strictly needed to secure sufficient statistically powerful end results. In addition, it would be worthwhile for pilot trials to evaluate methods of retaining patients before performing large-scale trials and to investigate, through qualitative evaluations, the reasons for patient drop-out.

Moreover, to successfully evaluate a reduction strategy, patient outcomes, such as pain severity and quality of life, should be assessed throughout the study to map the risks and benefits of opioid reduction strategies as these outcome measures are important topics in patient–doctor conversations on reducing opioids.^16^ Moreover, to increase generalisability of results, studies should be performed in more countries around the world.

Considering increasing waiting lists at pain clinics and rehabilitation centres, the positive outcome of Goodman *et al*,^20^ might inspire GPs to start with individually tailored opioid taper plans with patients who are receptive to that idea while taking the study’s limitations into consideration. Likewise, study results of Seal *et al*^23^ and Mehl-Madrona *et al*^30^ carefully point in the direction of multidisciplinary group therapeutic sessions with a role for non-pharmacological pain treatments. High drop-out rates in some studies^19^^,^^21^^,^^30^ suggest a need for close monitoring of patients when reducing their opioid treatment. Here, time is of the essence, something that is not always available in general practice. A role for nurse practitioners, as was proposed by Liebschutz *et al*,^29^ may be a solution; however, this will have to be investigated in more depth.

## Data

Systematic review protocol is available at PROSPERO 2021 CRD42021236399: https://www.crd.york.ac.uk/prospero/display_record.php?ID=CRD42021236399. Extraction forms are available from the corresponding author. The complete search strategy is available in Supplementary Box S1. The complete RoB 2.0 assessments and complete ROBINS-1 assessments are available in Supplementary Boxes S2 and S3, respectively.
